# End-of-life and bereavement support to families in cancer care: a cross-sectional survey with bereaved family members

**DOI:** 10.1186/s12913-024-10575-2

**Published:** 2024-02-01

**Authors:** Qëndresa Thaqi, Marco Riguzzi, David Blum, Simon Peng-Keller, Anja Lorch, Rahel Naef

**Affiliations:** 1 Institute for Implementation Science in Health Care, Faculty of Medicine, University of Zurich, Universitaetstrasse 84, 8006 Zurich, Switzerland; 2 Centre of Clinical Nursing Science, University Hospital Zurich, Zurich, Switzerland; 3 Competence Centre for Palliative Care, University Hospital Zurich, University of Zurich, Zurich, Switzerland; 4 Centre for Palliative Care, City Hospital Zurich, Zurich, Switzerland; 5 Spiritual Care, Faculty of Theology, University of Zurich, Zurich, Switzerland; 6 Medical Oncology and Hematology, University Hospital Zurich, Zurich, Switzerland

**Keywords:** Bereavement, Cancer, End-of-life, Family members, Palliative care, Quality of support

## Abstract

**Background:**

Losing a close other to cancer is an incisive experience that occurs after a long course of illness and intense family caregiving. Despite an evident need for family engagement and support and guidance on this, patients and family members may not receive the attention and support they need when a family unit is experiencing a disruption by death. A clear understanding of the quality of care that is currently provided and its ability to address family needs is necessary to improve end-of-life and bereavement support to families affected by cancer. The purpose of this study is to investigate the quality of support of end-of-life and bereavement care to families, their (un)met needs, grief experiences, and self-perceived health outcomes.

**Methods:**

A multi-center, cross-sectional observational survey study with family members (*n* = 35) whose close other died of cancer in a health institution or their own home in German-speaking Switzerland.

**Results:**

Bereaved family members were mostly satisfied with end-of-life care. Information on the grief process and services, and acknowledgment of their grief was experienced as helpful. Most coped with their grief drawing on family resources and exhibited resilience, but they reported unmet needs in relation to family togetherness and caregiving.

**Conclusion:**

This study with a small number of family members indicates that support provided to families across settings and illness trajectories is perceived as helpful, with specific needs related to family support. The findings suggest that improvements should focus on ensuring care that addresses the family as a unit and enables togetherness, mutual reflection, meaningful relationships, preparedness for death, resilience, and benefit-finding.

**Protocol registration:**

https://osf.io/j4kfh.

**Supplementary Information:**

The online version contains supplementary material available at 10.1186/s12913-024-10575-2.

## Background

Losing a close other to cancer is an incisive experience that occurs after a long illness course and intense family caregiving [1, 2]. Families’ experience of caregiving, communication about end-of-life, and support received as a family impact on their grief experience and psychosocial health [3–6], whereas families’ functioning and preparedness for death is associated with complicated grief responses [7, 8]. To promote family resilience and well-being over time and to prevent adverse family outcomes, there is a need to focus cancer care on the family as a unit to address their needs, soften their suffering, increase their preparedness for the loss, and facilitate living with the loss [9–12]. Evidence-based recommendations include assessing and addressing families’ needs early on, offering practical and emotional support to enable preferred caregiving roles, providing information and communication about end-of-life, promoting families’ preparedness for loss and ability to cope with death and dying as a family, offering health-promoting follow-up care to strengthen family resilience and well-being, and identifying and referring those at risk for complicated courses of bereavement to specialist services [12–17].

Despite the availability of evidence-based guidance [13, 14, 16, 17], patients and families may not receive the attention and support they need as a relational family unit that is about to experience a profound disruption by death [18–21]. Family members’ and cancer care professionals’ focus is often on the person with advanced cancer and care to family members usually ends with the patient’s death [16, 17, 22]. This means that families may not voice their own concerns and needs [23, 24]. At the same time health professionals may not recognize and proactively address families’ role and needs due to a lack of awareness, time, or priority-setting [25, 26]. Improvement in end-of-life and bereavement support to families affected by cancer has therefore been called for [27]. A clear understanding of the quality of care and its ability to address family needs is required. As part of the BEST CARE research project (Guideline-based end-of-life and BEreavement SupporT in Cancer CARE, https://osf.io/j4kfh), we assessed the type and quality of support that families received during end-of-life and in bereavement following the loss of a close other to cancer and explored their (un)met needs and bereavement outcomes.

## Methods

A cross-sectional survey was conducted with family members bereaved by cancer. The aims of this study were, first, to explore the quality of support that bereaved family members received at the end-of-life and in bereavement, the fulfillment of their needs during bereavement, and bereavement and health outcomes; second, to examine differences in these outcomes between different places of death (i.e., health institution vs. home care); and third, to examine the relationship between quality of support and bereavement outcomes.

### Setting and participants

The study was carried out in four hospitals and three home palliative care services in an urban area in German-speaking Switzerland. Family members were eligible to take part if they had been bereaved due to cancer for two to six months, had been a primary support person or carer, and were at least 18 years old. Family members who did not provide informed consent, did not possess sufficient German language skills to complete the questionnaire, were not cognitively able to understand or participate in the study, or had a self-reported mental health illness before their experience of loss, were excluded.

### Recruitment and data collection

Eligible participants were prospectively invited to participate by a clinician or service manager within the available recruitment period lasting from December 2021 to September 2022. First, they were informed about the study and received a study flyer. If they agreed, their contact details were communicated to the research team, who sent out a study information pack by email with a personalized link to the online questionnaire (REDcap; https://www.project-redcap.org), or by mail with a paper-pencil questionnaire. Participants were able to complete the questionnaire at a time of their convenience in their own home. Online questionnaires could be interrupted and then continued at a later time. If necessary, family members were reminded by phone or by email at three-week intervals.

The questionnaire had three sections focusing on [1] quality of support received during end-of-life and bereavement (56 items), [2] needs during bereavement (20 items), and [3] bereavement and health outcomes (17 items). Participants rated the questionnaire to be mostly understandable and easy to complete (*mdn* = 4, score range: 1 = totally disagree to 5 = totally agree, *n* = 34). They indicated that the length of the questionnaire was rather appropriate in terms of completion time (*mdn* = 4, score range: 1 = totally disagree to 5 = totally agree, *n* = 33). Psychometrically validated rating scales were used. In addition, three self-developed rating scales on self-perceived health were used after review for validity by researchers from nursing, bereavement care, family health, and psychology. Some of the measures were translated from English into German with permission using a forward-backward translation procedure [28] (see Table 1). Participating family members provided their demographics and proxy data on the demographics of the deceased and the circumstances of their death. A research assistant entered paper-pencil questionnaires into REDcap via direct entry forms.

**Table 1:** Overview of study endpoints and measures

Study endpoints	Measure	# Items	Score range	Score type	Cronbach’s α
**Quality of support**					
Quality of family support	ICEland Family Perceived Support Questionnaire (ICE-FPSQ)^1^	14	14–70	Sum	.92
	ICE-FPSQ Cognitive subscale	5	5–25	Sum	.86
	ICE-FPSQ Emotional subscale	9	9–45	Sum	.90
Quality of EoL care	CANadian Health Care EvaLuation Project– Bereavement version (CANHELP)^2*^	24	1–5	Mean	.93
Quality of BER support	Adapted Evaluation of Grief Support Services Tool^3^	11	Categorical	Raw	n/a
Support providers	Provider Support Assessment Tool^4^	7	Binary	Raw	n/a
**Needs**					
Fulfillment of needs	Needs Assessment of Family Caregivers– Bereaved to Cancer (NAFC-BvC)^5*^	20	0–16	Mean	>.76
**Bereavement and health outcomes**					
Coping	Meaning-Making questions^6^	3	1–5	Mean	n/a
Resilience	Brief Resilience Scale (BRS-6)^7^	6	1–5	Mean	.85
Grief intensity	Brief Grief Questionnaire (BGQ-5)^8^*	5	0–10	Sum	.75
Self-perceived health	Self-developed questions^9^	3	0-100	Raw	n/a

Note. * Translated with permission from English into German using a forward-backward translation procedure [22]; EoL = End-of-Life; BER = Bereavement; n/a = not applicable. The comprehensibility of the survey (*M* ± *SD*: 3.9 ± 0.9) and appropriateness of time requirements (*M* ± *SD*: 3.5 ± 1.1) were rated as rather high [i.e., 1 = low comprehensibility / appropriateness; 5 = high comprehensibility / appropriateness]

^1^ ICE-FPSQ = “ICEland Family Perceived Support Questionnaire” care [i.e., 14 = low quality of received support; 70 = high quality of received support], “ICE-FPSQ Cognitive subscale” care [i.e., 5 = low quality of received cognitive support; 25 = high quality of received cognitive support], “ICE-FPSQ Emotional subscale” care [i.e., 9 = low quality of received emotional support; 45 = high quality of received emotional support] [23, 24]

^2^ CANHELP = “CANadian Health Care EvaLuation Project– Bereavement version” [i.e., 1 = low satisfaction with EoL care; 5 = high satisfaction with EoL care] [25]

^3^ Questions assessing grief support evaluation, adopted with permission from the “Family Evaluation of Bereavement Services” (FEBS, Question 1) and the “Evaluation of Grief Support Services (EGGS, Questions 2–10) by the National Hospice and Palliative Care Organization, and Gallagher, Tracey, and Miller (2005) (Question 11) [26, 27]

^4^ Self-developed questions assessing received support by the different types of health professional groups: *“Who of the following health professional groups (i.e., nurses, physicians, medicinal assistant, chaplains, therapists, social workers, and others) provided support to you?”*, [i.e., yes / no]

^5^ NAFC-BvC = “Needs Assessment of Family Caregivers– Bereaved to Cancer” [i.e., 0 = high fulfillment; 16 = no fulfillment] [28]

^6^ “Meaning-making question”, “Benefit-finding question”, Identity change question” assessing meaning throughout the grieving process [i.e., 1 = no sense / low benefit / not different; 5 = good deal of sense / great benefit / very different] [30]

^7^ BRS-6 = “Brief Resilience Scale” [i.e., 1 = low resilience; 5 = high resilience] [31, 32]

^8^ BGQ-5 = “Brief Grief Questionnaire” [i.e., 0 = no impairment; 10 = high impairment] [33]

^9^ Self-developed questions assessing “State of health”: *“How is your current state of health?”*, “Well-being”: *“How is your current well-being?”*, and “Stress-level”: *“How is your current level of stress?”* [i.e., 0 = low state of health / low well-being / low stress-level; 100 = high state of health / high well-being / high stress-level]

### Study endpoints

#### Quality of support received as a family

The support families received from health professionals was assessed by the 14-item German version of the “ICEland Family Perceived Support Questionnaire” (ICE-FPSQ). It includes two subscales: “cognitive support” (5 items) and “emotional support” (9 items), which were rated on a 5-point Likert-type scale from 1 = *almost never* to 5 = *almost always*, resulting in a sum score of 14–70 overall, 5–25 for the cognitive, and 5–45 for the emotional support scale. This questionnaire showed high internal consistency (Cronbach’s above > 0.86) [29, 30].

In addition, we assessed which groups of health professionals (i.e., nurses, physicians, medicinal assistants, chaplains, therapists, social workers, and others) provided the support.

#### Quality of end-of-life care

To assess satisfaction with the end-of-life care, we used the 24-item “Canadian Health Care Evaluation Project - Bereavement Questionnaire” (CANHELP). Items were scored on a 5-point Likert-type scale ranging from 1 = *totally disagree* to 5 = *totally agree*. The CANHELP has high internal consistency, with a Cronbach’s alpha of 0.93 [31].

#### Quality of bereavement support

To obtain information on the type and quality of bereavement support, we adopted a selection of close-ended questions from the “Evaluation of Grief Support Services” (EGGS) by the National Hospice and Palliative Care Organization with permission [32], and one from a previous study by Tracey and Miller [33]. Questions pertained to grief information (i.e. “Were the following communicated to you after the death?”, “How useful was the information provided to you about…?”), follow-up support (e.g., “Was the number of telephone calls you received too few, about right, too many?”, “When you called for information or support, how did we do in getting you help as soon as you needed it?”), and questions about the overall quality of the service (i.e., “After the death, how well would you say our grief support services met your needs?”) and the skills of the person delivering the support (e.g., “How would you describe the skills of the person who provided grief support to you? S/he listened to me, understood me, was honest, was helpful” etc.).

#### Fulfillment of needs

To assess the (un)met needs among bereaved family members, we used the 20-item “Needs Assessment of Family Caregivers– Bereaved to Cancer” (NAFC-BvC). Each needsitem was rated twice on a 5-point Likert-type scale ranging from 0 = *not at all* to 4 = *extremely*, first for their importance and then for their satisfaction. Scores were calculated by multiplying the importance rating by the (reversed) satisfaction rating (scorerange: 0–16). The NAFC-BvC showed satisfactory internal consistency, with a Cronbach’s alpha of > 0.76 [34].

#### Coping

To measure coping with loss as proposed in the “Model of Meaning Reconstruction” [35, 36], three 5-point Likert-type scale questions were asked: “How much sense would you say have you made of the loss?” (i.e., 1 = *no sense*, 5 = *a good deal of sense*), “Despite the loss, have you been able to find any benefit from your experience of the loss?” (i.e., 1 = *no benefit*, 5 = *great benefit*), and “Do you feel that you are different, or that your sense of identity has changed, as a result of the loss?” (i.e., 1 = *no different*, 5 = *very different*).

#### Resilience

The 6-item German version of the “Brief Resilience Scale” (BRS-6) was used to examine the individual ability to recover from stress following the loss. Items were rated on a 5-point Likert-type scale ranging from 1 = *strongly disagree* to 5 = *strongly agree*. The BRS-6 has sufficient internal consistency, with a Cronbach’s alpha of 0.85 [37, 38].

#### Grief intensity

The 5-item “Brief Grief Questionnaire” (BGQ-5) was used to assess grief intensity. Items were scored on a 3-point Likert-type scale ranging from 0 = *not all* to 2 = *a lot*. This questionnaire exhibits sufficient internal consistency with a Cronbach’s alpha of 0.75 [39].

#### Self-perceived health

Three self-developed visual rating scales were used to assess self-perceived health, sense of well-being, and self-perceived stress: “How is your current health?” (i.e., 0 = *low state of health* to 100 = *high state of health*), “How is your current well-being?” (i.e., 0 = *low well-being* to 100 *= high well-being*), “How is your current level of stress?” (i.e., 0 = *low stress-level* to 100 = *high stress-level*).

### Data analysis

All rating variables were tested for normality using the Kolmogorov-Smirnov test and the Shapiro-Wilk test. Non-parametric statistical methods were used due to non-normal distribution and modest sample sizes to assess differences between the two places of death (two-tailed Mann-Whitney-U-test). To assess relationships between quality of support and bereavement outcomes, Spearman’s correlation was used and effect sizes were interpreted according to Cohen [40] with *r* = 0.10 indicating a small effect, *r* = 0.30 indicating a medium effect, and *r* = 0.50 indicating a large effect. If this relationship was significant, a regression analysis was performed to control for the potential influence of family member age, gender, relationship to deceased (partner / spouse vs. other), and time since death, as described in supplementary file 1. A p-value of < 0.05 was considered statistically significant. The statistical analyses were conducted using IBM SPSS version 28 and Stata/SE version 18 (regression analyses and approximations within scatterplots).

### Ethical considerations

The responsible Ethics Committee waived the need for approval (Req-2021-01054). Participants provided their informed consent at the beginning of the questionnaire.

## Results

### Participant characteristics

Of the 85 family members who received a questionnaire, 35 returned it (11 in electronic and 24 in written form, response rate of 41%). Nearly half of the participants were female (45.7%) (Table 2). Most of the participants were spouses (77.1%) or adult children (17.1%) of the deceased. The participants had lost their close other on average 3.5 months prior to a variety of cancer types. The deceased had been first diagnosed on average five years prior. Two thirds died at home. For most participants, the loss was expected (92.9%), and it predominantly occurred at the place preferred by the deceased (77.1%).

**Table 2:** Characteristics of family members and the deceased

Family member characteristics	*N* = 35	Metrics
**Age** in years *M* ± *SD*	33	62.9 ± 12.6
**Female gender** [yes] *n* (%)	35	16 (45.7)
**Religion***n* (%)	34	
Catholic		25 (73.5)
Undenominational		9 (26.5)
**Birthplace in Middle-Europ*e****n* (%)	35	29 (82.9)
**Highest degree***n* (%)	35	
Vocational training		17 (48.6)
Diploma		10 (28.6)
University degree (≥ Bachelors’ Degree)		8 (22.9)
**Work status***n* (%)	33	
Employed		13 (39.4)
Retired		20 (60.6)
**Employment level** [0-100%] *M* ± *SD*	13	89.2 ± 18.9
**Co-habiting with deceased***n* (%)	35	28 (80.0)
**Relation to deceased: the answering family member is…***n* (%)	35	
Spouse / partner		27 (77.1)
Daughter / son		6 (17.1)
Mother / father		1 (2.9)
Sister / brother		1 (2.9)
**Caregiver during illness trajectory** [yes] *n* (%)	35	33 (94.3)
**Time since loss** in months *M* ± *SD*	35	3.5 ± 1.3
**Death was expected by answering family member** [yes] *n* (%)	35	29 (92.9)
**Present at time of death** [yes] *n* (%)	35	30 (85.7)
**Own health problems** [yes] *n* (%)	34	3 (8.8)
**Importance of spirituality in own life** [0-100] *M* ± *SD*	35	35.9 ± 28.7
**Characteristics of the deceased**	***n***	**Metrics**
**Age** in years *M* ± *SD*	35	70.1 ± 9.5
**Female gender** [yes] *n* (%)	35	19 (54.3)
**Living situation***n* (%)	35	
Alone		3 (8.6)
Spouse / partner		26 (74.3)
Family (> 2 persons)		6 (17.1)
**Has children** [yes] *n* (%)	35	28 (71.4)
**Time between diagnosis and death** in years *M* ± *SD*	34	5.3 ± 5.0
**Primary place of care** (last month) *n* (%)	30	
Health institution^1^		8 (26.7)
Home care^1^		22 (73.3)
**Place of death***n* (%)	35	
Health institution^2^		13 (37.1)
Home care^2^		22 (62.9)
**Death at preferred place** [yes] *n* (%)	35	27 (77.1)

Note. *M* = mean, *SD* = standard deviation

^1^ Place of care: health institution: hospital (*n* = 4), hospice (*n* = 1), care home (*n* = 3); home care: own home (*n* = 21), family members’ home (*n* = 1)

^2^ Place of death: health institution: hospital (*n* = 7), hospice (*n* = 2), care home (*n* = 4); home care: own care (*n* = 21), family members’ home (*n* = 1)

### Quality of support

The quality of support that families received was rated with a median score of 50.0 (ICE-FPSQ, score range: 14–70). More specifically, family members rated the received cognitive support with a median score of 20.0 (ICE-FPSQ Cognitive support, score range: 5–25) and the emotional support with a median score of 34.0 (ICE-FPSQ Emotional support, score range: 9–45). Families whose close other had died at home on average had higher results in all three scores than those whose close other had died in an institution. However, these tendencies were not statistically significant (see Table 3).

**Table 3:** Quality of support, needs, bereavement and health outcomes

	All (*n* = 35)		Health Institution (*n* = 13)		Home care (*n* = 22)		p-value*
**Quality of support**	*n*		*n*		*n*		
**Quality of family support (ICE-FPSQ)** ^1^ *sum (range)*	31	50.0 (14.0–70.0)	9	35.0 (14.0–70.0)	22	55.0 (23.0–70.0)	0.094
ICE-FPSQ Cognitive support	31	20.0 (5.0–25.0)	9	12.0 (5.0–25.0)	22	20.5 (7.0–25.0)	0.094
ICE-FPSQ Emotional support	31	34.0 (9.0–45.0)	9	25.0 (9.0–45.0)	22	34.5 (16.0–45.0)	0.174
**Quality of EoL care (CANHELP)** ^2^ *mdn (range)*	26	4.2 (2.2-5.0)	9	4.1 (3.3-5.0)	17	4.3 (2.2-5.0)	0.958
**Support providers***n* (%)							
Nurses	35	34 (97.1)	13	13 (100.0)	22	21 (95.5)	
Physicians	34	18 (52.9)	12	7 (53.3)	22	11 (50.0)	
Medical assistants	35	28 (80.0)	13	12 (92.3)	22	16 (72.7)	
Chaplains	34	15 (44.1)	12	6 (50.0)	22	9 (40.9)	
Therapists	34	4 (11.8)	12	1 (7.7)	22	3 (13.6)	
Social workers	35	7 (20.0)	13	3 (23.1)	22	4 (18.2)	
Others	34	9 (26.5)	12	1 (7.7)	22	8 (36.4)	
**Fulfillment of needs (NAFC-BvC)** ^3^ *mdn (range)*	*n*		*n*		*n*		
Taking part in your usual social/recreational activities (item 1)	33	3.0 (0.0–6.0)	11	3.0 (0.0–3.0)	22	2.5 (0.0–6.0)	0.693
Talking to others who have lost a loved one to cancer (item 2)	32	0.5 (0.0–9.0)	12	1.0 (0.0–6.0)	20	0.5 (0.0–9.0)	0.774
Reorganizing roles among family members (item 3)	35	0.0 (0.0–16.0)	13	0.0 (0.0–6.0)	22	1.0 (0.0–16.0)	0.287
Taking care of bills (item 4)	33	0.0 (0.0–12.0)	11	0.0 (0.0–4.0)	22	0.0 (0.0–12.0)	0.418
Talking with other people about cancer (item 5)	33	2.0 (0.0–6.0)	11	2.0 (0.0–3.0)	22	2.0 (0.0–6.0)	0.895
Dealing with emotional distress of other family members (item 6)	32	0.0 (0.0–9.0)	11	0.0 (0.0–6.0)	21	0.0 (0.0–9.0)	0.584
Finding meaning out of your experience with his/her cancer (item 7)	32	0.0 (0.0–6.0)	11	0.0 (0.0–6.0)	21	0.0 (0.0–4.0)	0.815
Getting help with your household activities (item 8)	34	0.0 (0.0–12.0)	12	0.0 (0.0–12.0)	22	0.0 (0.0–6.0)	0.901
Understanding medical and/or insurance coverage (item 9)	34	0.0 (0.0–12.0)	12	0.0 (0.0–9.0)	22	0.0 (0.0–12.0)	0.873
Being satisfied with your relationship with family members / friends (item 10)	33	3.0 (0.0–9.0)	12	3.0 (0.0–9.0)	21	3.0 (0.0–6.0)	0.811
Taking care of your own health (item 11)	34	3.0 (0.0–12.0)	12	3.0 (0.0–8.0)	22	3.0 (0.0–12.0)	0.986
Dealing with lifestyle changes (item 12)	33	2.0 (0.0–16.0)	12	1.0 (1.0–9.0)	21	2.0 (0.0–16.0)	0.618
Dealing with your emotional distress (item 13)	34	3.0 (0.0–16.0)	13	1.0 (0.0–9.0)	21	3.0 (0.0–16.0)	0.400
Getting help from others to take time for yourself (item 14)	34	0.0 (0.0–12.0)	12	0.0 (0.0–12.0)	22	1.0 (0.0–9.0)	0.363
Meeting your personal needs (item 15)	32	2.0 (0.0–6.0)	10	2.5 (0.0–6.0)	22	2.0 (0.0–4.0)	0.795
Getting together with family / friends (item 16)	32	3.0 (0.0–9.0)	10	3.0 (0.0–4.0)	22	1.5 (0.0–9.0)	0.219
Taking time off from work (item 17)	32	0.0 (0.0–12.0)	10	0.0 (0.0–3.0)	22	0.0 (0.0–12.0)	0.646
Understanding/navigating the health care system (item 18)	32	2.0 (0.0–8.0)	10	2.5 (0.0–8.0)	22	1.0 (0.0–8.0)	0.889
Helping other family members find meaning out of his/her cancer (item 19)	33	0.0 (0.0–8.0)	11	0.0 (0.0–6.0)	22	0.0 (0.0–8.0)	1.000
Getting legal paperwork done (item 20)	32	2.5 (0.0–16.0)	11	3.0 (0.0–16.0)	21	2.0 (0.0–16.0)	0.639
**Bereavement and health outcomes**							
**Coping** ^4^ *mdn (range)*							
Meaning-Making	34	1.5 (1.0–5.0)	12	1.0 (1.0–4.0)	22	2.5 (1.0–5.0)	0.444
Benefit-finding	34	4.0 (1.0–5.0)	12	4.0 (1.0–5.0)	22	3.0 (1.0–5.0)	0.231
Identity change	33	2.0 (1.0–4.0)	12	2.4 (1.0–4.0)	21	2.0 (1.0–4.0)	0.518
**Resilience (BRS-6)** ^5^ *mdn (range)*	34	3.5 (2.3-5.0)	12	3.8 (2.8-5.0)	22	3.4 (2.3-5.0)	0.136
**Grief intensity (BGQ-5)** ^6^ *mdn (range)*	34	4.0 (0.0–10.0)	12	4.0 (0.0–7.0)	22	5.0 (0.0–10.0)	0.309
**Self-perceived health** ^7^ *mdn (range)*							
Self-perceived health	33	81.0 (28.0-100.0)	12	90.0 (50.0-100.0)	21	75.0 (28.0-100.0)	0.228
Sense of well-being	33	66.0 (21.0-100.0)	12	67.50 (40.0-100.0)	21	66.0 (21.0-100.0)	0.567
Self-perceived stress	33	40.0 (0.0–90.0)	12	24.5 (0.0–85.0)	21	60.0 (0.0–90.0)	0.122

Note. *mdn* = median, * *p*-value of a two-sided Mann-Whitney U-test between the groups health institution and home-care

^1^ ICE-FPSQ = “ICEland Family Perceived Support Questionnaire”, score range: 14–70; high score = high quality of received support, “ICE-FPSQ Cognitive subscale”, score range: 5–25, high score = high quality of received cognitive support, “ICE-FPSQ Emotion subscale”, score range: 9–45, high score = high quality of received emotional support

^2^ CANHELP = “CANadian Health Care EvaLuation Project– Bereavement version”, score range: 1–5, high score = high satisfaction in EoL care

^3^ NAFC-BvC = “Needs Assessment of Family Caregivers– Bereaved to Cancer”, score range: 0–16, high score = no fulfillment

^4 “^Meaning-making question”, score range: 1–5; high score = having made a good deal of sense of the death, “Benefit finding question”, score range: 1–5, high score = great benefit, “Identity change”, score range: 1–5, high score = very different sense of identity

^5^ BRS-6 = “Brief Resilience Scale”, score range: 1–5; high score = high resilience

^6^ BGQ-5 = “Brief Grief Questionnaire”, score range: 0–10; low score = low grief reaction

^7^ Self-developed questions, score range: 0-100; high score = high self-perceived health / high sense of well-being / high self-perceived stress

Bereaved family members rated the quality of end-of-life care with a median score of 4.2 (CANHELP, score range: 1–5), with no statistically significant difference between places of death.

Families reported to have received the following types of bereavement care: acknowledgement of their individual grief experience (78.8%, *n =* 33), validation of grief as a normal reaction (65.6%, *n* = 32), information on grief and loss (61.8%, *n* = 34), and follow-up calls (51.6%, *n* = 34). Availability of information on coping with grief (57.6%, *n* = 33) and memorial services (48.3%, *n* = 29) were considered helpful by about half of the families, information on follow-up visits (33.3%, *n* = 30) and support groups (34.4%, *n* = 32) were less often seen as helpful. More than half of the families rated grief support and educational information as sensitive to their cultural and/or spiritual background (73.1%, *n* = 26). Grief services were perceived to match personal needs (74.1%, *n* = 27), and were experienced as compassionate (89.7%, *n* = 29).

On average, families received support from four different professional groups, most often from nurses (97.1, *n* = 34), followed by medical assistants (80.0%, *n* = 28), physicians (52.9%, *n* = 18), and chaplains (44.1%, *n* = 15). A majority (at least *n* = 24–25, 89.3%) perceived providers to be highly skilled in listening, showing understanding, and in being trustful, accepting, honest, and helpful.

### Needs

Most of the assessed needs were reported as fulfilled (NAFC-BvC; score range: 0–16) in the sense of low relevance or complete satisfaction (Table 3). The least fulfilled needs were “taking part in your usual social / recreational activities” (item 1), “being satisfied with your relationship with family members / friends” (item 10), “taking care of your own health” (item 11), “dealing with your emotional distress” (item 13), and “getting together with family / friends” (item 16). There were no statistically significant differences in scores between places of death.

### Bereavement and health outcomes

In terms of coping, bereaved family members reported low levels of meaning-making of the loss (*mdn* = 1.5, score range:1–5) and of identity change (*mdn* = 2.0, score range: 1–5). In contrast, families rated their benefit-finding as high (*mdn* = 4.0, score range: 1–5). No statistically significant differences between places of death were found.

On average, bereaved families showed a median score of 3.5 in their resilience response (BRS, score range: 1–5). There was no significant difference across places of death.

Grief intensity was rated with medium values (*mdn* = 4.0, score range: 0–10). No statistical significance difference between places of death was found.

Family members indicated their self-perceived health with a median score of 81.0 (score range: 0-100), their sense of well-being with a median score of 66.0 (score range: 0-100), and their self-perceived stress with median score of 40.0 (score range: 0-100) (see Table 3).

### Relationships between quality of support and bereavement outcomes

**Fig. 1:**
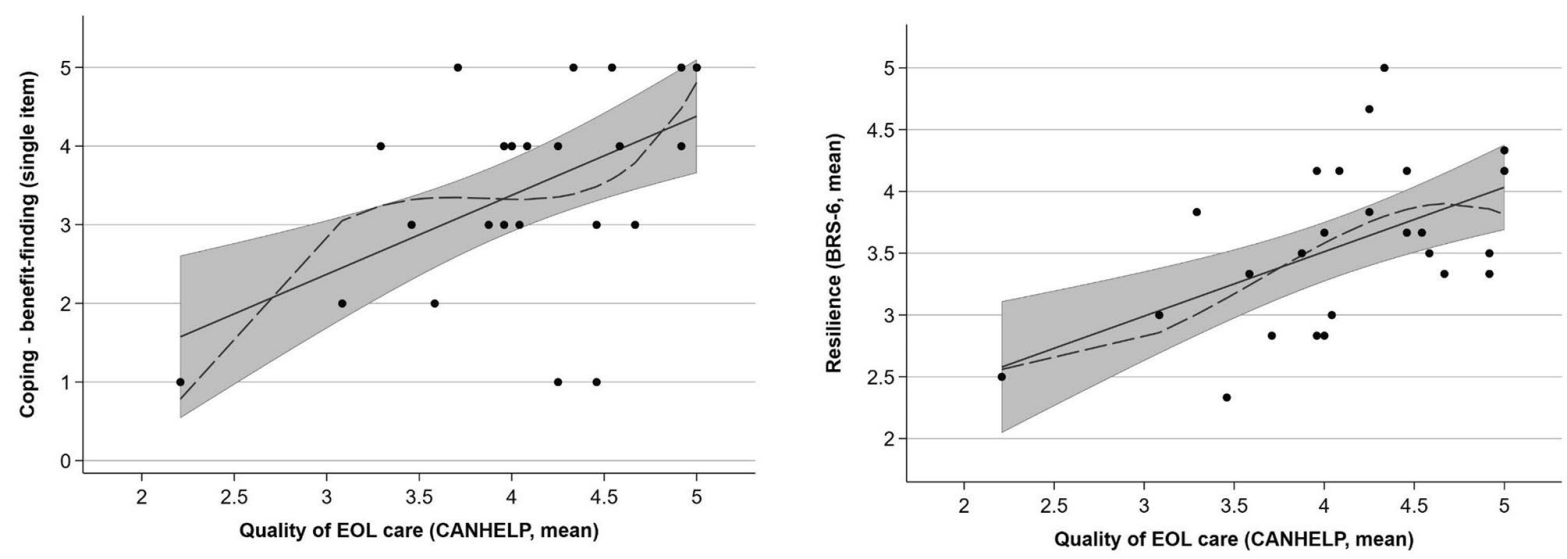
Scatterplots of coping (benefit-finding) vs. quality of end-of-life care and resilience (BRS-6) vs. quality of end-of-life care *Note. Added to each scatterplot are a third-degree fractional polynomial approximation (generalized linear model, GLM) and a linear approximation with a 95% confidence interval. Scatterplots of all pairings of quality of support vs. bereavement outcomes are provided in Supplementary file*2. CANHELP = “CANadian Health Care EvaLuation Project– Bereavement version”, score range: 1–5, high score = high satisfaction in EoL care. BRS-6 = “Brief Resilience Scale”, score range: 1–5; high score = high resilience. EOL = End-of-Life Care.

Statistically significant, positive correlations were found between quality of end-of-life care (CANHELP) and resilience (BRS-6) (*r* = 0.49, *p* = 0.015, *n* = 25), and between quality of end-of-life care and benefit-finding (*r* = 0.47, *p* = 0.019, *n* = 25) (see Table 4; Fig. 1 / Supplementary file 2 for scatterplots). These relationships proved to be robust when potential influencers were controlled for using multiple regression analyses (see Table 5). The quality of end-of-life care mean score (CANHELP; range 2.2-5 in the regression sample, *n* = 25) had a positive relationship with the benefit-finding (range 1–5) that was nearly 1:1 (incremental effect = 0.933, *p* = 0.025), i.e. an increase in quality of end-of-life care mean score by 1 corresponds to an increase in the benefit-finding by 0.933 on average. The quality of end-of-life care mean score (CANHELP) also had a positive relationship with the resilience mean score (BRS-6; range 2.3-5 in the regression sample, *n* = 25), whereby an increase in the quality of end-of-life score by 1 implies on average an increase in the resilience score by 0.426 (*p* = 0.012). Family member age was negatively associated with resilience (incremental effect per year = -0.032, *p* = 0.004).

**Table 4:** Relationship between quality of support and bereavement outcomes (Spearman’s correlation coefficient)

	Bereavement outcomes									
**Quality of support**	**Coping** ^3^ Meaning-Making	*n*	**Coping** ^3^ Benefit-finding	*n*	**Coping** ^3^ Identity change	*n*	**Resilience** ^4^ BRS-6	*n*	**Grief intensity** ^5^ BGQ-5	*n*
**Quality of family support** (ICE-FPSQ)^1^	0.12	30	0.17	30	− 0.12	29	0.13	30	− 0.16	30
ICE-FPSQ Cognitive support^1^	0.17	30	0.26	30	0.33	29	0.29	30	− 0.30	30
ICE-FPSQ Emotional support^1^	0.09	30	0.12	30	− 0.05	29	0.08	30	− 0.08	30
**Quality of EoL care** (CANHELP)^2^	0.16	25	0.47*	25	− 0.08	25	0.49*	25	− 0.35	25

Note. * *p* < 0.05

^1^ ICE-FPSQ = “ICEland Family Perceived Support Questionnaire” care, the “ICE-FPSQ Cognitive subscale”, and the “ICE-FPSQ Emotional subscale”

^2^ CANHELP = “CANadian Health Care EvaLuation Project– Bereavement version”

^3^ Coping = “Meaning-making question”, “Benefit-finding question”, and “Identity change question”

^4^ BRS-6 = “Brief Resilience Scale”

^5^ BGQ-5 = “Brief Grief Questionnaire”

**Table 5:** Fractional logistic regression models of benefit-finding and resilience (*n* = 25)

Regressors / measures of model fit	Benefit-finding (coping), single item (1–5)			Resilience (BRS-6), mean score (1–5)		
	AME^a^	p^b^	95%-CI^c^	AME^a^	p^b^	95%-CI^c^
**Full model:**						
*Quality of end-of-life care (CANHELP) (mean)*	0.933*	*0.025*	*[0.117; 1.749]*	0.426*	*0.012*	*[0.093; 0.759]*
*Time since death to survey completion (months)*	*0.112*	*0.468*	*[-0.191; 0.415]*	*0.103*	*0.309*	*[-0.095; 0.300]*
*Family member age (years; range 35–90, mean = 61)*	*0.008*	*0.748*	*[-0.043; 0.059]*			
*From minimum age (35 years) to under 50 years*				*0.057*	*0.138*	*[-0.018; 0.132]*
*From 50 years to maximum age (90 years)*				-0.032**	*0.004*	*[-0.053; -0.010]*
Family member is the partner / spouse of the deceased (yes)	0.002	0.997	[-1.199; 1.203]	0.309	0.355	[-0.346; 0.964]
Family member is female (vs. male)	-0.084	0.885	[-1.232; 1.063]	-0.073	0.698	[-0.440; 0.294]
**BIC-optimized model**:						
*Quality of end-of-life care (CANHELP) (mean; range 1–5)*	0.976***	*0.000*	*[0.476; 1.477]*	0.503***	*0.000*	*[0.279; 0.728]*
**Likelihood-based pseudo-R**^**2**^**measures of model fit**:						
Full model:						
Nagelkerke’s R^2^, Cox & Snell’s R^2^, McFadden’s R^2^	0.153	0.112	0.090	0.085	0.062	0.049
BIC-optimized model:						
Nagelkerke’s R^2^, Cox & Snell’s R^2^, McFadden’s R^2^	0.146	0.107	0.085	0.041	0.030	0.023

*Note. Continuous variables in italics.* *p < 0.05, **p < 0.01, ***p < 0.001. ^*a*^*Average marginal effect.*^*b*^*p-value of type one error.*^*c*^*Limits of 95% confidence interval.*

CANHELP = “CANadian Health Care EvaLuation Project– Bereavement version”, score range: 1–5, high score = high satisfaction in EoL care.

BRS-6 = “Brief Resilience Scale”, score range: 1–5; high score = high resilience.

## Discussion

This cross-sectional study with a small sample of family members recently bereaved due to cancer found that families are mostly satisfied with end-of-life care. About two thirds indicated to receive bereavement support reflective of evidence-based recommendations, such as acknowledgement, validation, and information on dying, grief and available support [41]. Support was received from many different health professionals who had– according to family members high interpersonal skills [42, 43], with nurses and medical office assistants being a core support source for them.

Needs related to family togetherness and relationships, social activities, self-care, and dealing with emotional stress remained most often unmet, reinforcing the need to better support families across settings [9, 18, 44]. Our study shows that families felt less well supported, particularly in relation to their cognitive dimensions (i.e., information, education, reflection opportunities within the family), which has also been previously shown in cancer care setting [18, 45].

We observed some statistically non-significant differences between places of death. Those whose close other had died at home seemed to feel better supported as a family unit than those whose close other had died in hospital or another institutional setting. At the same time, families indicated to have higher unmet needs related to their caregiving role, such as reorganization of roles, getting help from others, dealing with lifestyle changes, or emotional distress, compared to those whose close other died in an institution [11, 46]. In contrast, getting together as a family, navigating the healthcare system, and getting legal paperwork done were more prominent unmet needs among those whose close other died in a hospital. Given the small sample size and low statistical power, these findings need to be interpreted with caution, but suggest that support needs may differ according to place of death. Larger studies with representative samples are needed to verify findings within a cancer care context, potentially extending to other life-limiting illnesses. Overall, a focus on the family as a relational system with support needs related to family management of caregiving, loss, and bereavement, in addition to individual foci on patient or family members, is called for [23, 45, 47–50].

As previously reported, we also identified an association between high satisfaction with end-of-life support with resilience and benefit-finding, which stresses the importance of quality end-of-life care [3, 7, 8, 51–53]. Higher age was associated with lower resilience. Participants indicated moderate grief intensity, high resilience, and benefit-finding. Many family members reported lower levels of meaning-making of the loss, which may be due to the fact that they participated in the study within the first months after their loss [54, 55]. Those caring for their dying family member at home indicated higher stress-levels than those whose close other died in institution, which may be related to the burden of care [4, 8].

### Study limitations

Our study is limited by a small, non-representative sample. Partners and those with home care were overrepresented compared to the general population. It is possible that participants represent a more satisfied and resilient group among bereaved family members who are well able to live with their loss in close interactions with their social network [56]. The recruitment of participants in a small geographical area and the limited diversity of the study participants might have introduced a selection bias that may affect the generalizability of the results. Due to low statistical power, lack of statistical significance does not amount to sufficient evidence of the absence of an effect / difference. While we mostly used psychometrically validated instruments, we also relied on three self-developed questions and non-validated German versions. Hence, our findings need to be interpreted with caution. Nevertheless, this cross-sectional observational study offers important insights about the actual quality of support in end-of-life and bereavement care, state of health, and (un)met needs perceived from bereaved family members.

## Conclusion

This study reveals high satisfaction with end-of-life support and needs fulfillment based on a small sample of family members recently bereaved to cancer, which is associated with resilience and benefit-finding. The perception of the quality of bereavement care received as a family unit by health professionals in general and during bereavement is more modest, particularly for those whose close other died in an institutional setting such as hospital, suggesting a need for better support structures and care provided by interprofessional teams. While our study focuses on families bereaved due to cancer, our findings may have relevance for other populations, such as families bereaved following a persistent illness. Study findings suggest that improvements should focus on ensuring care that supports the family as a unit and enables togetherness, mutual reflection, meaningful relationships, strong support networks, preparedness for death, family resilience, and benefit-finding. Based on this survey, the quality of and specific needs for bereavement support are less conclusive, partly due to measurement issues, and require further investigation with representative samples to better understand quality of bereavement care [57].

### Electronic supplementary material

Below is the link to the electronic supplementary material.

Supplementary Material 1

## Data Availability

Data are available on reasonable request from the authors.

## Abbreviations

BEST CARE: BEreavement SupporT in Cancer CARE
REDcap: Electronic Data Capture System
ICE-FPSQ: ICEland Family Perceived Support Questionnaire
CANHELP: Canadian Health Care Evaluation Project - Bereavement Questionnaire
EGGS: Evaluation of Grief Support Services
NAFC-BvC: Needs Assessment of Family Caregivers– Bereaved to Cancer
BRS-6: Brief Resilience Scale
BGQ-5: Brief Grief Questionnaire
IBM SPSS: Statistical Package for the Social Sciences
Mmdn: median
r: Correlation coefficient
p: Type-one error probability
n: Number of observations
