# Supplementary material for: End-of-life and bereavement support to families in cancer care: a cross-sectional survey with bereaved family members
Source: BMC Health Serv Res. 2024 Feb 1;24:155. doi: 10.1186/s12913-024-10575-2 (PMC10832212; doi:10.1186/s12913-024-10575-2)
Supplement: Supplementary file 1 — Supplementary Material 1 [file 12913_2024_10575_MOESM1_ESM.pdf]

Supplementary files

End-of-life and bereavement support to families in cancer care: A cross-sectional survey with bereaved family members

Qëndresa Thaqi, Marco Riguzzi, David Blum, Simon Peng-Keller, Anja Lorch, Rahel Naef

Supplementary file 1
Regression analysis of the relationship of quality of end-of-life care (CANHELP) with coping (meaning-making) and resilience (BRS-6)

Supplementary file 2
Scatterplots of quality of support vs. bereavement outcomes

Supplementary file 1 | Regression analysis of the relationship of quality of end-of-life care (CANHELP) with coping (meaning-making) and resilience (BRS-6)

Multiple regression analyses were performed using Stata/SE (version 18) to test for possible biases in the significant correlations shown in Table 4 between (1) benefit-finding (coping) and quality of end-of-life care (CANHELP) ($n = 25$) and (2) resilience (BRS-6) and quality of end-of-life care (CANHELP) ($n = 25$). Due to the type of outcome and the small number of observations, fractional logistic rating scale regressions (see, e. g., (1, 2)) with robust standard errors (3-5) were performed to ensure validity. Model fit was assessed by the likelihood-based pseudo coefficients of determination by Nagelkerke (6), Cox and Snell (7), and McFadden (8). Multicollinearity between the predictors was tested by the variance inflation factor (9), resulting in a maximum of 3.04. In the model of resilience (BRS-6), family member age had a significant non-linear relationship with the logit, which was modeled by a spline function with a node at 50 years of age (see, e. g., Hosmer, Lemeshow (11)). The results were calculated as average marginal effects (AME) on the scale of the respective outcome, 1 to 5. The regression results are shown in Table 5. In both models, backward optimization of Bayes' information criterion (BIC) resulted in the elimination of all predictors except quality of end-of-life care (CANHELP).

Ordinary least squares (OLS) regressions with robust standard errors were also conducted for the same models, showing no significant indications of a violation of the OLS assumptions according to the Tukey-Anscombe plot, normal QQ plot, and scale location plot. Influential observations within the small sample were assessed by their leverage and Cook's distance (10), resulting in no values of Cook's distance exceeding one. OLS results did not differ fundamentally from those of the fractional logistic models, meaning that the same predictors were significant in each model ($p < 0.05$) irrespective of the estimation method, and that the significant estimates differed by ±30.4% at most (AME vs. OLS point estimate).

References

1. Papke LE, Wooldridge JM. Econometric Methods for Fractional Response Variables with an Application to 401(K) Plan Participation Rates. Journal of Applied Econometrics. 1996;11:619-32.
2. Studer R, Winkelmann R. Econometric Analysis of Ratings – with an Application to Health and Wellbeing. Swiss Society of Economics and Statistics. 2017;153(1):1-13.
3. Eicker F. Asymptotic Normality and Consistency of the Least Squares Estimators for Families of Linear Regressions. The Annals of Mathematical Statistics. 1963;34(2):447-56.
4. Huber PJ. The behavior of maximum likelihood estimates under nonstandard conditions. Proceedings of the Fifth Berkeley Symposium on Mathematical Statistics and Probability. 1967;5:221-33.

5. White H. A Heteroskedasticity-Consistent Covariance Matrix Estimator and a Direct Test for Heteroskedasticity. Econometrica. 1980;48(4):817-38.
6. Nagelkerke NJD. A Note on a General Definition of the Coefficient of Determination. Biometrika. 1991;78(3):691-2.
7. Cox DR, Snell EJ. Analysis of Binary Data. 2nd Edition ed. London: Chapman & Hall; 1989.
8. McFadden D. Conditional logit analysis of qualitative choice behavior. In: Zarembka P, editor. Frontiers in econometrics. New York: Academic Press; 1974. p. 105-42.
9. Snee R. Origins of the Variance Inflation Factor as Recalled by Cuthbert Daniel. Snee Associates; 1981.
10. Cook RD. Detection of Influential Observations in Linear Regression. Technometrics. 1977;19(1):15-8.
11. Hosmer DW, Lemeshow S, Sturdivant RX. Applied Logistic Regression. Third edition ed: Wiley; 2013.

Supplementary file 2 | Scatterplots of quality of support vs. bereavement outcomes

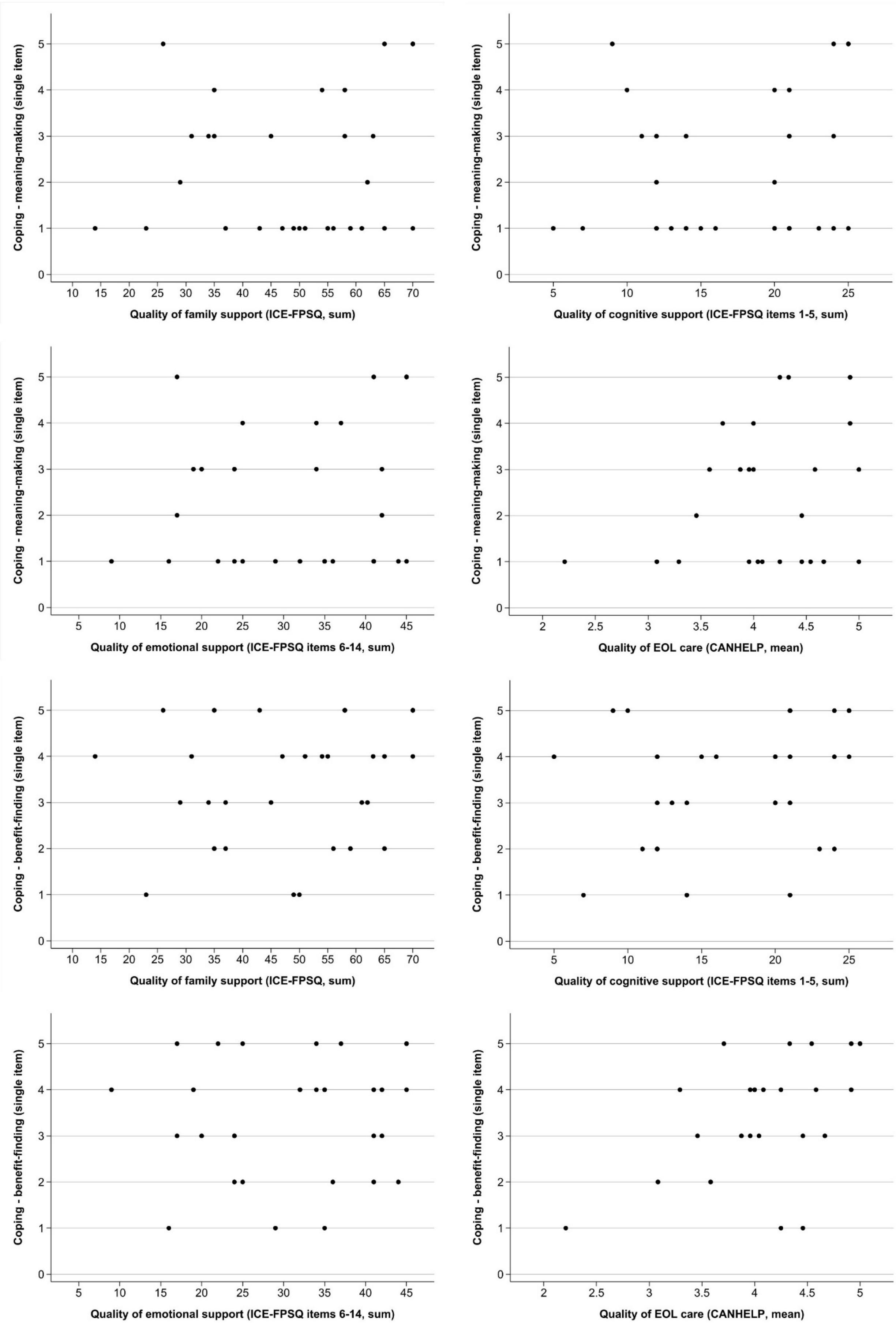

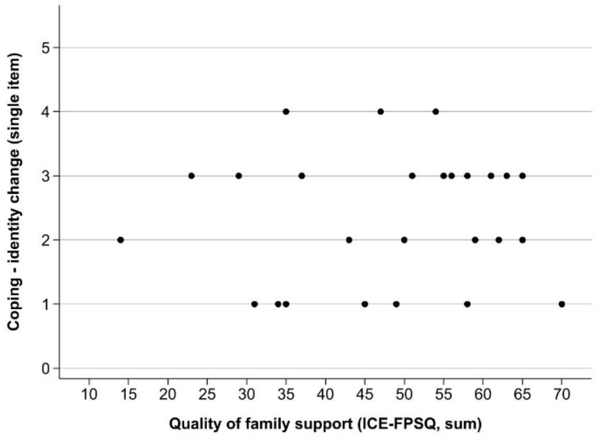
Coping - identity change (single item)
Quality of family support (ICE-FPSQ, sum)

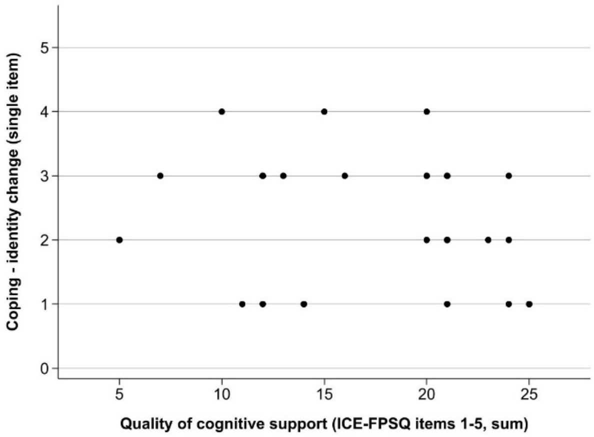
Coping - identity change (single item)
Quality of cognitive support (ICE-FPSQ items 1-5, sum)

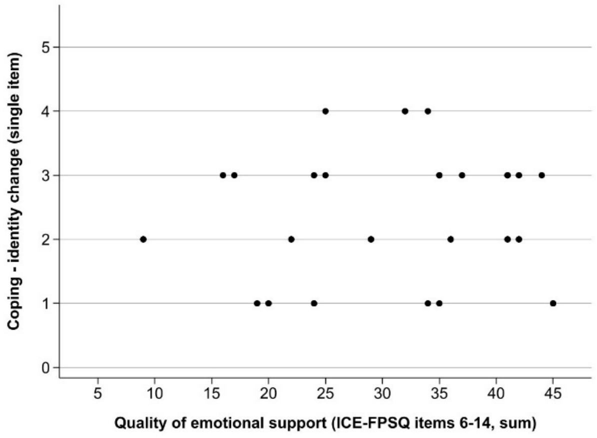
Coping - identity change (single item)
Quality of emotional support (ICE-FPSQ items 6-14, sum)

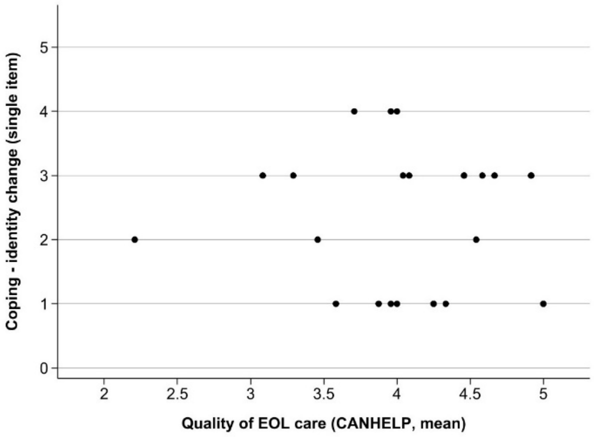
Coping - identity change (single item)
Quality of EOL care (CANHELP, mean)

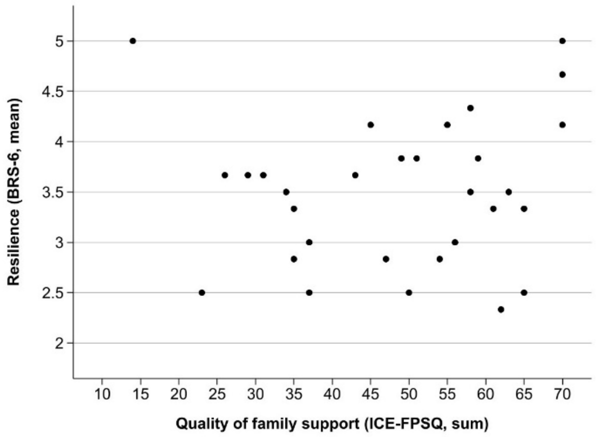
Resilience (BRS-6, mean)
Quality of family support (ICE-FPSQ, sum)

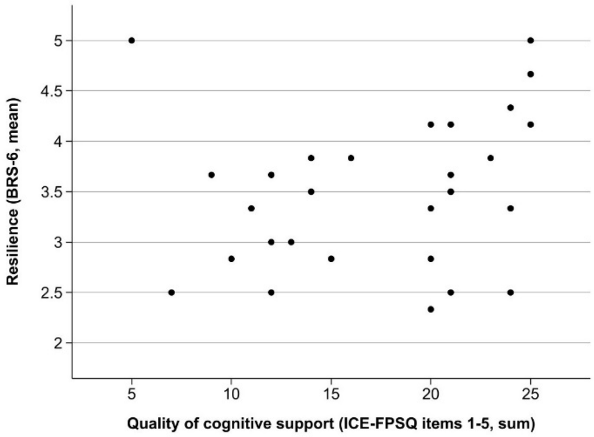
Resilience (BRS-6, mean)
Quality of cognitive support (ICE-FPSQ items 1-5, sum)

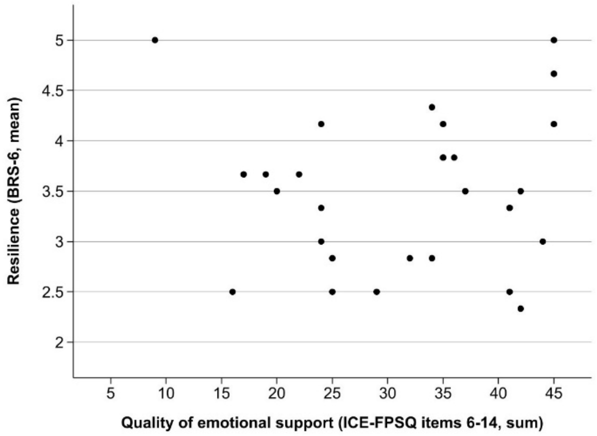
Resilience (BRS-6, mean)
Quality of emotional support (ICE-FPSQ items 6-14, sum)

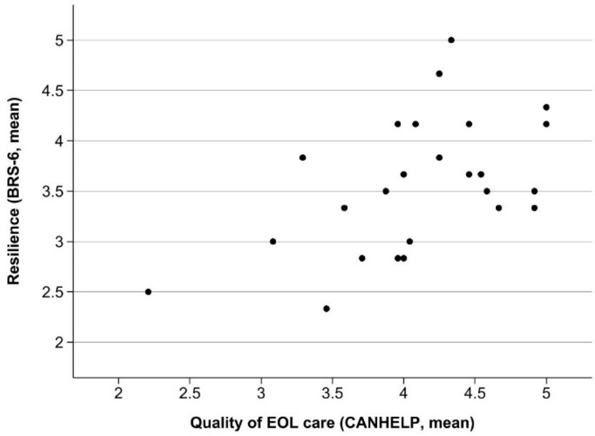
Resilience (BRS-6, mean)
Quality of EOL care (CANHELP, mean)

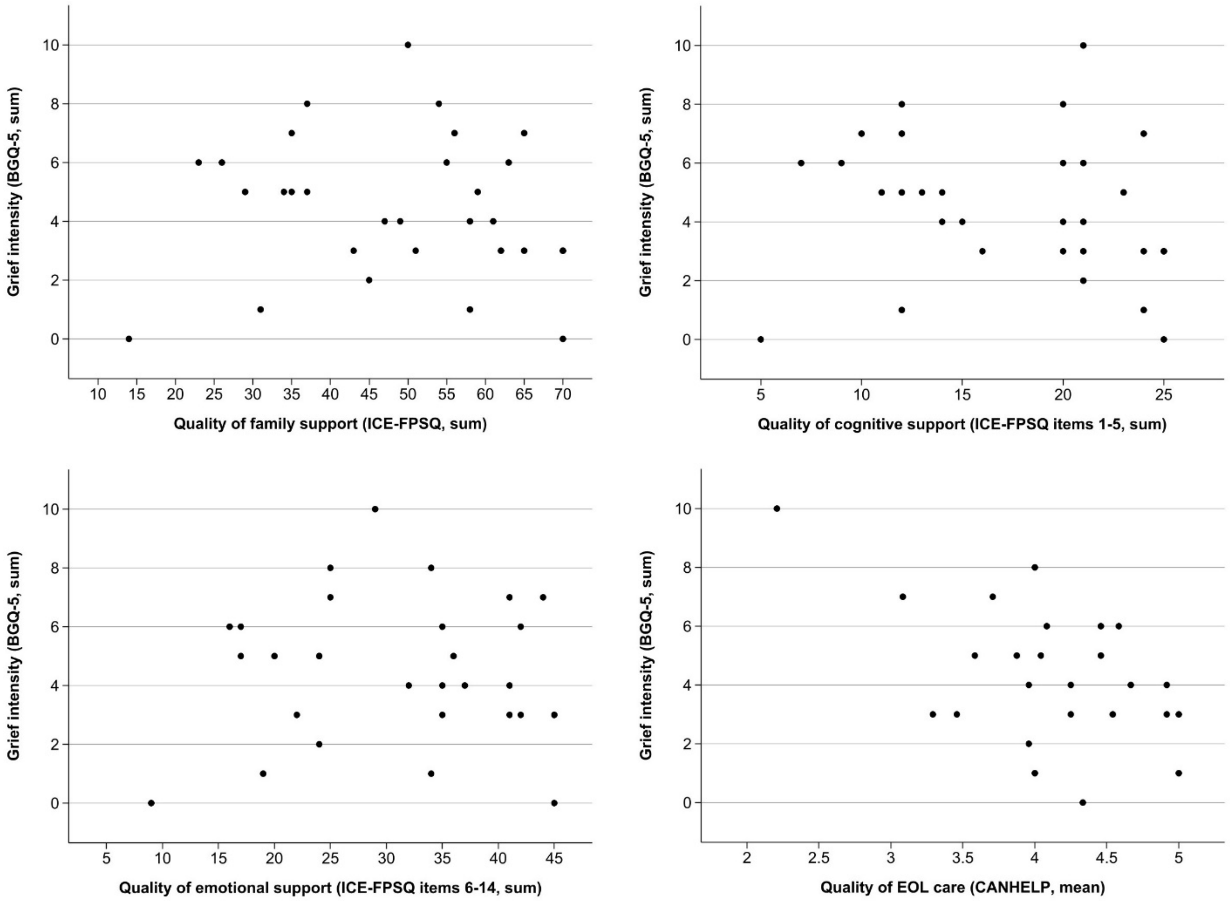

Grief intensity (BGQ-5, sum)
Quality of family support (ICE-FPSQ, sum)
Grief intensity (BGQ-5, sum)
Quality of cognitive support (ICE-FPSQ items 1-5, sum)
Grief intensity (BGQ-5, sum)
Quality of emotional support (ICE-FPSQ items 6-14, sum)
Grief intensity (BGQ-5, sum)
Quality of EOL care (CANHELP, mean)
